# Increased emission intensity can compensate for the presence of noise in human click-based echolocation

**DOI:** 10.1038/s41598-021-81220-9

**Published:** 2021-01-18

**Authors:** J. G. Castillo-Serrano, L. J. Norman, D. Foresteire, L. Thaler

**Affiliations:** Department of Psychology, Durham University, Science Site, South Road, Durham, DH1 3LE UK

**Keywords:** Perception, Human behaviour

## Abstract

Echolocating bats adapt their emissions to succeed in noisy environments. In the present study we investigated if echolocating humans can detect a sound-reflecting surface in the presence of noise and if intensity of echolocation emissions (i.e. clicks) changes in a systematic pattern. We tested people who were blind and had experience in echolocation, as well as blind and sighted people who had no experience in echolocation prior to the study. We used an echo-detection paradigm where participants listened to binaural recordings of echolocation sounds (i.e. they did not make their own click emissions), and where intensity of emissions and echoes changed adaptively based on participant performance (intensity of echoes was yoked to intensity of emissions). We found that emission intensity had to systematically increase to compensate for weaker echoes relative to background noise. In fact, emission intensity increased so that spectral power of echoes exceeded spectral power of noise by 12 dB in 4-kHz and 5-kHz frequency bands. The effects were the same across all participant groups, suggesting that this effect occurs independently of long-time experience with echolocation. Our findings demonstrate for the first time that people can echolocate in the presence of noise and suggest that one potential strategy to deal with noise is to increase emission intensity to maintain signal-to-noise ratio of certain spectral components of the echoes.

## Introduction

Echolocation is the ability to use reflections of sound, typically from self-produced sonar emissions, to obtain spatial information about the environment. It has been well described in bats and marine mammals^1–4^, but humans can echolocate as well^5–7^. Echolocating bats rely on the transmission and reception of acoustic signals in order to interact effectively with the environment, as observed in their ability to obtain food and move about in dark spaces. Bats adapt their echolocation calls based on the acoustic circumstances, emitting louder signals to boost the intensity of weak echoes^8^ and maintain their echolocation behaviour in background noise^9^. To respond to increasing background noise levels, echolocating bats produce louder calls^9,10^. It has been suggested that bat’s vocal modifications in the presence of noise are an effort to improve signal-to-noise ratio in conditions of increasing auditory masking^11^, i.e. effectively bats attempt to reduce auditory masking of their echolocation signals. Bats may also increase the duration of their calls in response to background noise^11^, though the relationship between this modification and changes in signal-to-noise ratio are less clear. The possibility arises that just as observed in echolocating bats, adaptations to the intensity of echolocation emissions may be a strategy used in human echolocation behaviour in background noise in order to improve or maintain signal-to-noise ratios.

To our knowledge, no research to date has explored the ability of humans to echolocate in acoustically challenging situations. Therefore, in the present study we investigated if humans can accurately detect a sound reflecting surface in the presence of background noise, and if the intensity of echolocation emissions (i.e. clicks) is systematically adapted in this context. We expected that, if adjustments of emission intensity are designed to maintain or improve signal-to-noise ratio, we should find a systematic relationship between the resulting strength of the echo relative to the background noise.

We tested these ideas in people who were blind and had experience in echolocation, as well as in people who were blind or sighted and had no prior experience in echolocation. People with experience in echolocation make very brief clicks with spectral content in higher frequency bands, and these acoustic features of clicks are related to behavioural advantages in terms of echo perception^12,13^. Thus, to ensure that spectro-temporal features of emissions did not present a potential confound across participant groups we used a paradigm where participants listened to binaural recordings of echolocation sounds, and where emission intensity of these recordings was adjusted using an adaptive staircase procedure. We found that emission intensity had to systematically increase to compensate for weaker echoes relative to background noise, consistent with the idea that increased emission intensity serves to improve or maintain signal-to-noise ratios. Consistent with this idea a spectral analysis also revealed that in 4-kHz and 5-kHz frequency bands spectral power of echoes exceeded spectral power of noise by 12 dB in all conditions. In addition, the effects were the same across all participant groups, suggesting that long-time experience with echolocation is not required for this effect to occur.

## Methods

All procedures followed the British Psychological Society code of practice and the World Medical Association’s Declaration of Helsinki. The experiment had received ethical approval by the Ethics Advisory Sub-Committee in the Department of Psychology at Durham University (Ref 16/19). All participants gave written informed consent to take part in this study. For blind participants, all materials were provided in accessible format and locations to sign were indicated through tactile markers. Participants were compensated either with participant pool credits or £10/h.

### Participants

Three blind expert echolocators (EEs, mean age: 43.3, SD: 8.1; 1 female), eight blind people new to echolocation (BCs, mean age: 53.3, SD:12.7; 2 female) and 20 sighted (normal or corrected to normal vision) people new to echolocation (SCs, mean age: 22.8, SD: 4.2, 17 female) took part. Please see Table 1 for details of blind participants. All subjects reported to have normal hearing and for all blind participants this was confirmed using pure tone audiometry (250–8000 Hz; Hughson Westlake; Interacoustics AD629 audiometer, Interacoustics, Denmark).

**Table 1:** Details of blind participants who took part in the study.

Participant code	Age at time of testing	Gender	Degree of vision impairment at time of testing	Cause of vision impairment and age at onset	History of use of echolocation
EE1	52	M	Total blindness	Retinoblastoma; onset at birth; enucleation at age 13 months	Daily; since early childhood/no exact age remembered
EE2	36	M	Total blindness	Severe childhood glaucoma; gradual since birth	Daily; since 12 years old
EE3	42	F	Total blindness	Retinoblastoma; onset at birth; enucleation at 22 months	Daily since age 30 years
BC1	67	M	Bright light perception	Leber's Amaurosis; from birth	–
BC2	33	M	Total blindness	Retinopathy of prematurity	–
BC3	67	M	Total blindness	Retinopathy of prematurity	–
BC4	56	F	Yes, total right eye; left eye tunnel vision	Accident at age 44; vision loss due to damage along optic nerve/chiasm	–
BC5	54	M	Bright light perception	Retinitis Pigmentosa; birth; progressive	–
BC6	46	M	Bright light perception	Ocular albinism; birth; progressive	–
BC7	64	F	Total blindness	Eyes did not develop; birth	–
BC8	40	M	Total blindness	Unknown cause from birth; detached retinas leading to total blindness at age 7	–

*EE* expert echolocator, *BC* blind control.

### Experimental sounds

#### Sound emissions

**Figure 1:**
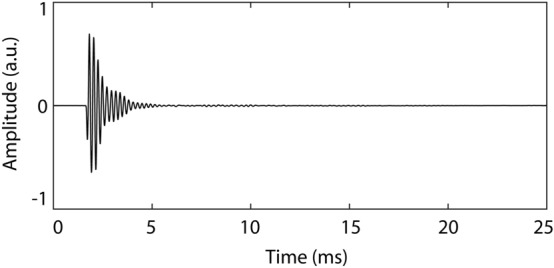
Waveform plot (left channel only) of recording of click emission made in front of the speaker. a.u. – arbitray units.

Click emissions were constructed by first creating a 10-ms 4.5-kHz sinusoid and then multiplying all values up until the first half period by 0.6. Then, all values after the first 1.5 periods were multiplied by the output of the decaying exponential function y = e^−6x^, where x is a series of linear equally spaced values between 0 and 1 that is equal in length to the number of values in the sinusoid between the first 1.5 periods and its end. Figure 1 shows a waveform plot of the resulting click sound as recorded in front of the speaker (see Recording Equipment and Setup). This type of sound (a sinusoid multiplied by a decaying exponential) has been suggested previously to be a good approximation of the waveform created by a human echolocator’s mouth click^13,14^ and has been used successfully as an artificial emission in previous tasks of echolocation^12,15^. As background noise we used white noise recordings taken from the RSG-10_Noise database^16^ (http://www.steeneken.nl/7-noise-data-base/).

#### Recording equipment and setup

**Figure 2:**
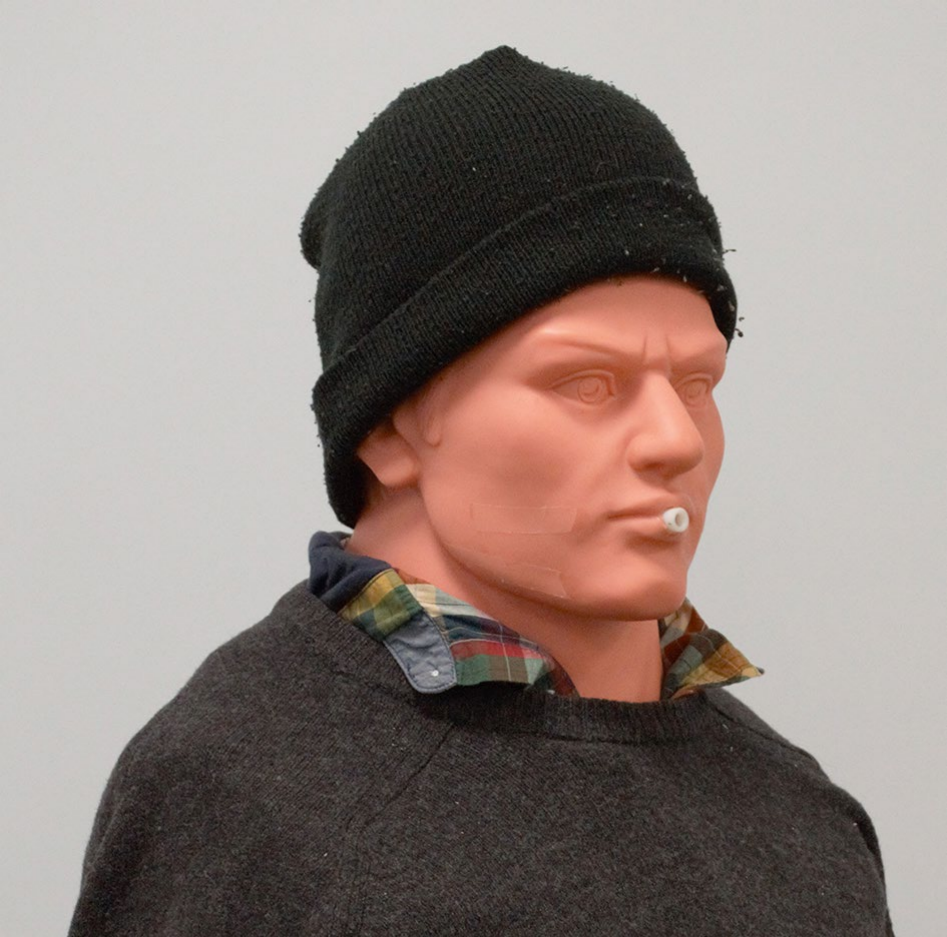
Picture of the manikin used to make binaural recordings. Anthropometric details of this manikin have been published elsewhere^12^. The center of the speaker used to generate emissions was placed at the mouth of the manikin (marked by a small tube).

All sound recordings were made in a sound-insulated and echo-acoustic dampened room (approx. 2.9 m × 4.2 m × 4.9 m; 24-dBA noise floor) lined with foam wedges (cut-off frequency 315 Hz). Binaural sound recordings were made at a sampling rate of 96 kHz and resolution of 24-bit using a portable digital recorder (Tascam DR-100 MK2, TEAC Corporation, Japan) and in-ear microphones (Bruel & Kjaer model 4101, Denmark) placed in the ears of a manikin. The manikin was custom-made, consisting of a torso and head made of high-density foam covered with soft plastic having a skin like texture, and wearing a woollen jumper and hat. Anthropometric details of this manikin have been published elsewhere^12^. A picture of the manikin is provided in Fig. 2.

**Figure 3:**
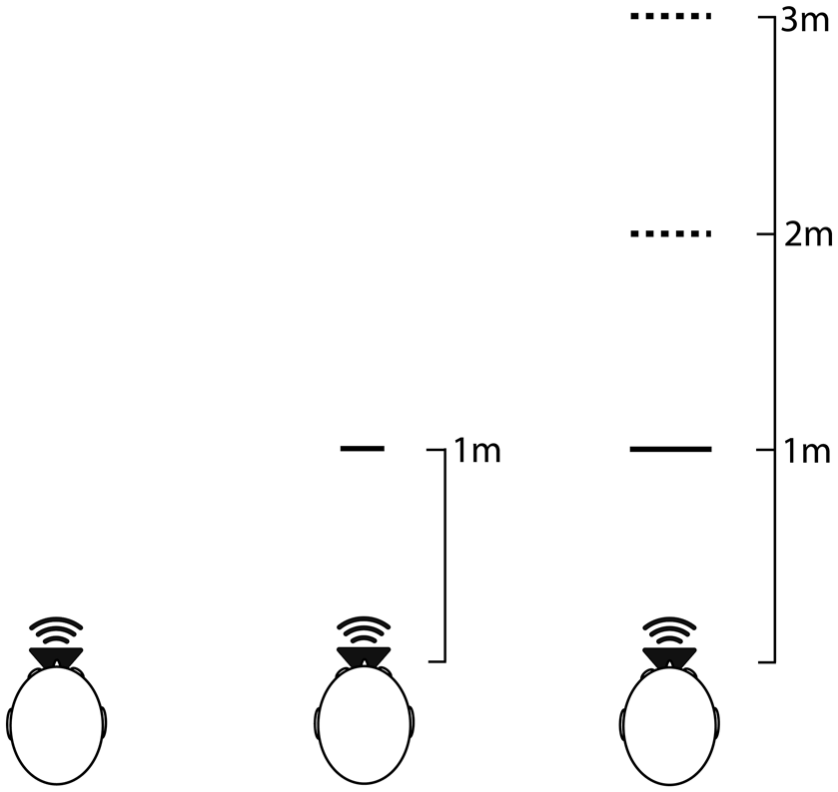
Illustration (top view) of recording setup for the various echolocation sound conditions used in the experiment. Echolocation sounds were recorded in an empty space (object absent; left), in the presence of a 17.5-cm diameter disk at 1-m distance (middle) and in the presence of a 26.5-cm diameter disk that could be placed at 1 m, 2 m, or 3 m (right). In all conditions the disk was placed so that its round, flat side faced the manikin. Here (in the top view) the disk is illustrated as a horizontal line.

**Figure 4:**
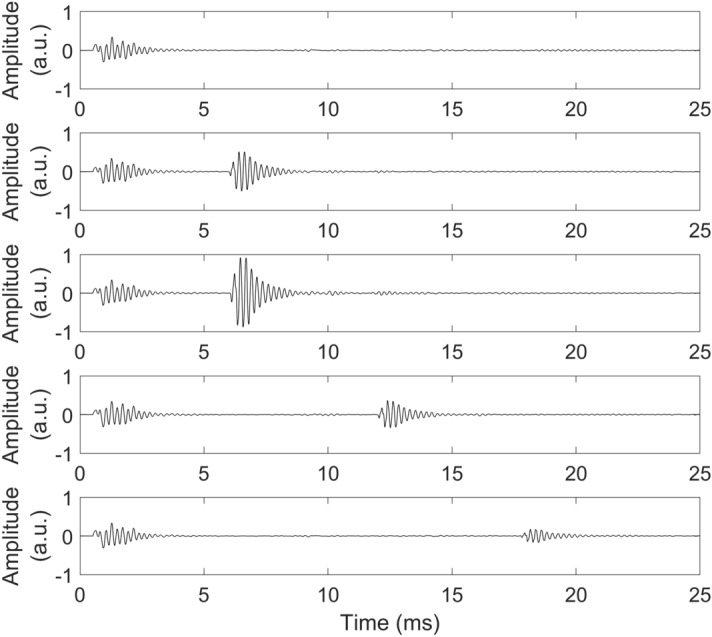
Waveform plots (left channel only) of echolocation sound recordings used during the experiment. From top to bottom: Object Absent, 17.5-cm disk at 1 m, 26.5-cm disk at 1 m, 26.5-cm disk at 2 m, 26.5-cm disk at 3 m. a.u. – arbitray units.

Artificially generated echolocation emissions were played by a loudspeaker (Fostex FE103En) mounted on a metal pole (1 cm diameter) placed at the mouth of the manikin. The loudspeaker was driven by a Dell Latitude E7470 laptop (Intel Core i56300U CPU 2.40 GHz, 8 GB RAM, 64-bit Windows 7 Enterprise) through a USB Soundcard (Creative Sound Blaster X-Fi HD Sound Card; Creative Technology Ltd., Creative Labs Ireland, Dublin, Ireland) and amplified by a Kramer 900 N Stereo Power Amplifier (Kramer Electronics Ltd., Jerusalem, Israel). Sounds were played using Matlab R2015b (The Mathworks, Natick, MA). Level of amplification in all electronic equipment was held constant for the recording of all sounds across all conditions. Sound recordings for echolocation sounds corresponded to the experimental conditions in our study and the conditions were based on different combinations of object sizes and distances. The objects were 0.8-mm thick disks of either 17.5 or 26.5 cm diameter, made from plywood covered with matte emulsion paint. During echolocation recordings the 17.5-cm disk could be placed at 1 m facing the manikin, and the 26.5-cm disk could be placed at 1, 2 and 3 m, respectively. In all conditions the disk was placed so that its round, flat side faced the manikin. In addition, we made echolocation recordings when no disk was present in the room. Figure 3 illustrates the recording set-up. Figure 4 shows waveform plots (left channel only) of echolocation sound recordings used during the experiment. In Fig. 4 one can see that in some cases the sound pressure of the echo is higher than that of the emission measured by the microphone. The relative intensity difference between echo and emission for echolocation sounds used in our experiment (illustrated in Fig. 4) for the 17.5-cm target at 1 m, and 26.5-cm target at 1, 2 and 3-m distance were − 3.48, − 8.64, − 0.08 and 5.15 dB respectively. These values were calculated based on root mean square (RMS) intensity for 3 ms measured from onset of each sound. The relationship between the two sound pressures is affected by the reflector used (i.e. louder echoes for larger and closer reflectors), but also by directional characteristics of the speakers used (i.e. sound energy is primarily projected towards the front, and less towards the side and back, where binaural microphones were located).

### Task and procedure

#### Setup and apparatus

Participants were tested in a sound-insulated and echo-acoustic dampened room (approx. 3 m × 2.5 m × 3.3 m) in Durham University Psychology department. Sounds were played to participants through in-ear headphones (Etymotic Research ER4B MicroPro) driven by a PC (Intel Core i56600 CPU 3.30 GHz, 16-GB RAM, 64-bit operating system, × 64-based processor, Windows 10 Home) through a USB Soundcard (Creative Sound Blaster X-Fi HD Sound Card; Creative Technology Ltd., Creative Labs Ireland, Dublin, Ireland). Participants sat upright and gave their response using a keyboard. Participants who were not fully blind wore a blindfold. All experiments were programmed in Matlab R2015b (The Mathworks, Natick, MA) and Psychtoolbox (v3.0.12)^17^. Sounds were played to participants at a level at which the sound file with the highest peak intensity was presented at 80 dB SPL.

#### Behavioural paradigm

All subjects in this study completed a series of training and test sessions. In training sessions participants were made familiar with the echolocation task without any background noise. In test sessions, participants completed the same task as done during training, but in the presence of background noise. The level of the emission was adjusted in an adaptive staircase procedure, with the level of background noise remaining constant. The goal of test sessions was to determine the level of the emission that was required for participants to perform the echolocation task at a certain accuracy (71% correct) in the presence of background noise.

During the echolocation task, on each trial, subjects heard two consecutive echolocation recordings separated by a gap of 1750 ms. One recording contained a click and echo, whereas the other recording contained a click only. The presentation order of the two sounds was random on each trial and participants pressed the ‘z’ key on the computer keyboard to indicate that they perceived the echo in the first sound and pressed the ‘m’ key if they perceived the echo in the second sound.

The order in which participants completed the four experimental conditions (i.e. 17.5-cm disk at 1 m, 26.5-cm disk at 1 m, 2 m or 3 m) was counterbalanced across participants, i.e. subjects were assigned to one of four different presentation orders of the four experimental conditions. All participants first completed training sessions of any one test condition, followed by the test session for that same condition, before performing any of the remaining experimental conditions. It typically took participants 3 h to complete all training and testing sessions. To prevent fatigue, breaks were provided to all participants in between experimental conditions, including the option to complete different experimental conditions on separate days.

#### Training procedure and sounds

During training sessions no background noise was present and the two echolocation sounds were separated by a silent gap of 1750 ms. In the first part of the training, subjects completed blocks of 40 trials with feedback until they obtained at least 90% accuracy. Participants heard a high pitch tone (1200 Hz) when they gave a correct response and a low pitch tone (600 Hz) when they gave an incorrect response. Once participants had achieved at least 90% accuracy, they completed an additional block of 40 trials without any feedback. If participants reached at least 90% accuracy without feedback, they would then proceed to testing. If their performance without feedback dropped below 90%, they were trained again with feedback. On average, subjects reached this criterion after 1.32 training blocks with feedback (SD: 0.60) and after 1.02 training blocks without feedback (SD: 0.07). Average accuracy across all conditions and groups was 98.9% (SD: 2.15), and accuracy was the same across conditions (F(3,84) = 1.501; *p* = 0.220; η_p_^2^ = 0.051) and groups (F(2,28) = 1.555; *p* = 0.229; η_p_^2^ = 0.1) and there was also no significant interaction effect (F(6,84) = 0.347; *p* = 0.91, η_p_^2^ = 0.024).

#### Testing procedure and sounds

During testing sessions background noise was present so that each sound contained an echolocation sound as well as white noise. White noise was a 1250-ms segment cut at random positions from the white noise recording taken from the RSG-10 Noise database^16^ (http://www.steeneken.nl/7-noise-data-base/). The first 250 ms of each noise segment were linearly ramped, and the echolocation sound was presented in the middle of the remaining 1000 ms of noise. Thus, the temporal sequence for each test trial was as follows: 250 ms linearly ramped noise, 1000 ms noise (including echolocation sound), 500 ms silence, 250 ms linearly ramped noise, 1000 ms noise (including echolocation sound). The level of the echolocation sound (i.e. signal) was determined in a 2-up-1-down adaptive staircase procedure in steps of 3 dB, with the level of the white noise (i.e. noise) held constant. Specifically, the signal-to-noise ratio (SNR) was defined as the ratio (in dB) of the RMS intensity of the 10 peak values of the emission relative to the 10 peak values of the white noise. The intensity of the emission decreased by 3 dB after two consecutive correct responses, and increased by 3 dB after one incorrect response. Four randomly interleaved adaptive staircases were run in each test session. Each of these four staircases had a different starting SNR value (i.e. − 20, − 10, 0 and + 10 dB). A staircase terminated after 14 response reversals (i.e. from correct to incorrect or vice versa).

### Data analysis

#### Analysis of behavioural data

Psychometric curves were fitted to data for each experimental condition across all four staircases. Specifically, using Matlab R2015b (The Mathworks, Natick, MA) and the Palamedes toolbox^18^ we fitted psychometric functions (cumulative normal, with threshold and slope as free parameters) using a maximum likelihood criterion to describe proportion correct as a function of SNR. We then chose the SNR threshold at which the function returned a proportion correct of 0.75 as the threshold estimate, i.e. the SNR at which people are expected to obtain 75% accuracy in their responses.

Subsequently, SNR data were analyzed using mixed model ANOVA with ‘experimental condition’ as the within-subject factor and ‘group’ (SC, BC and EE) as the between-subject factor. If sphericity could not be assumed, Greenhouse Geisser Correction (GG) was applied. Since group sizes were unbalanced, we additionally analyzed group effects for each condition separately using non-parametric Kruskal–Wallis tests.

#### Additional acoustic analyses

SNR values at threshold were defined as the intensity of the emission relative to the intensity of the white noise, and this chosen based on the literature in bats^8–11^, which addresses emissions. Yet, the intensity of the emission alone is not sufficient for participants to perform this task—they must discriminate the presence/absence of the echo. If intensity of the emission is adjusted with the goal to maintain or improve SNR of echoes relative to background noise, then we should expect to see equal SNRs of the echo to noise at threshold across all conditions tested. Thus, to investigate how people’s performance relates to this aspect of the sounds, we used emission-based SNR values to also calculate the SNR for the echo intensity at threshold for each participant and condition. The SNR of the echo to noise was defined in the same way as the SNR of the emission to noise.

Furthermore, we also used SNRs in the overall intensity domain to calculate power of noise, clicks and echoes in the spectrum. This way we could determine any systematic differences in SNR by spectral frequency. This analysis was performed for each condition separately. SNR in the intensity domain was averaged across participants prior to calculating power in the spectrum.

## Results

**Figure 5:**
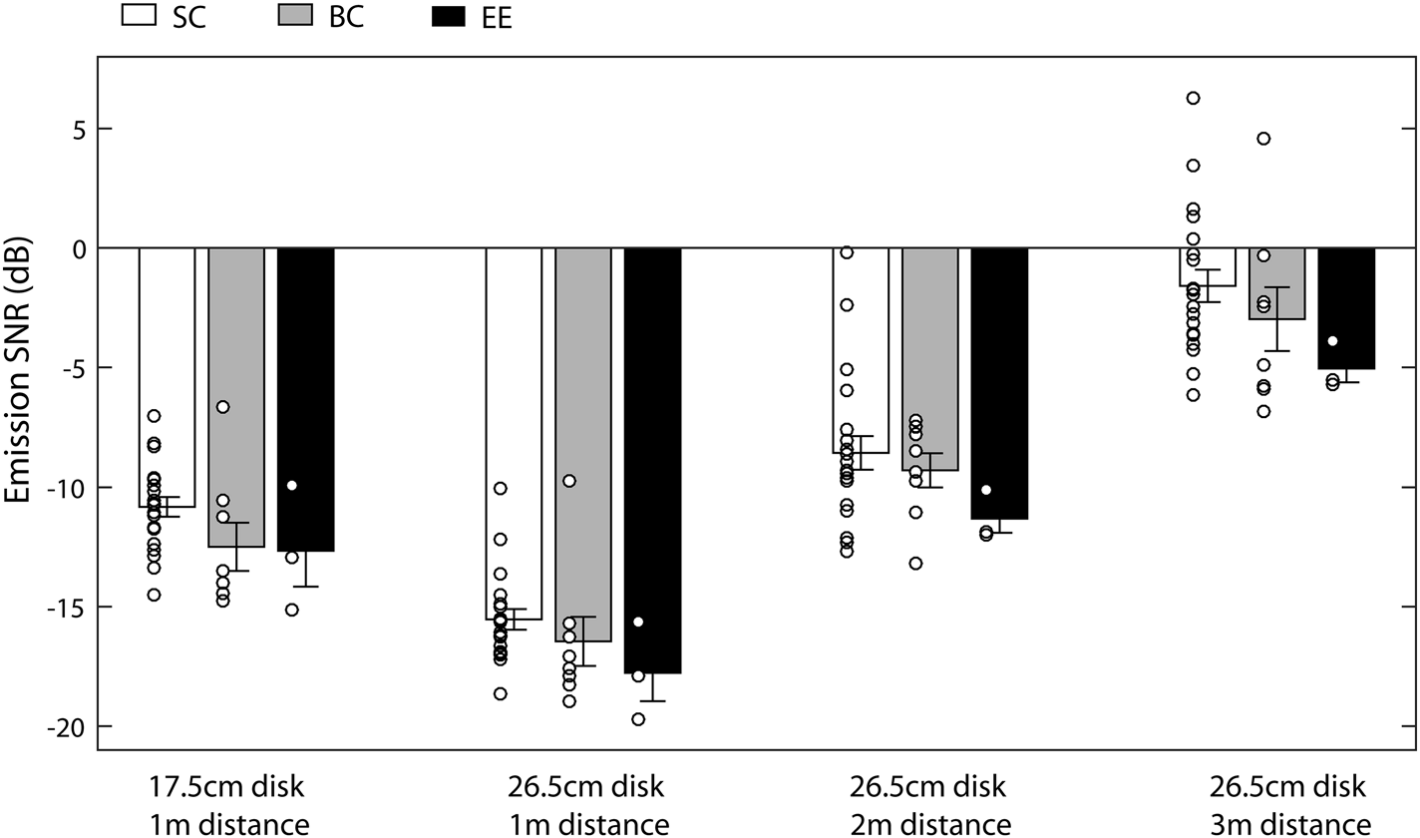
Emission SNRs for the different conditions and participant groups (SC – sighted controls; BC – blind controls; EE – expert echolocators). Bars represent means and error bars represent standard error of the mean across participants. Circles represent individual participants.

Emission SNR values for the different conditions and groups are shown in Fig. 5. It is evident that SNRs differ across conditions, but are similar across all participant groups. Specifically, SNRs are lowest, i.e. participants tolerated highest levels of background noise relative to the intensity of the emission, in conditions where the 26.5-cm disk was presented at 1 m. SNRs increase, i.e. people tolerate less noise, as distance increases (i.e. 26.5-cm disk at 1 m vs. 2 m vs. 3 m), or as the sound reflecting surface becomes smaller (i.e. 17.5-cm vs. 26.5-cm disk at 1 m). Consistent with these observations the ANOVA showed a significant main effect of ‘experimental condition’ (F_GG_(2.357, 65.996) = 158.391, *p* < 0.001; η_p_^2^ = 0.85), whereas the interaction between ‘experimental condition’ and ‘group’ was non-significant (F(4.714, 65.996) = 0.364, *p* = 0.862; η_p_^2^ = 0.025) and also the main effect of ‘group’ was non-significant (F(2,28) = 2.524, *p* = 0.098; η_p_^2^ = 0.153). Also non-parametric Kruskal Walllis tests did not reveal any effect of ‘group’ for any of the test conditions (17.5 cm at 1 m: *X*^2^(2,31) = 4.789; *p* = 0.091; 26.5 cm at 1 m: *X*^2^(2,31) = 5.527; *p* = 0.063; 26.5 cm at 2 m: *X*^2^(2,31) = 3.261; *p* = 0.196; 26.5 cm at 3 m: *X*^2^(2,31) = 4.729; *p* = 0.094). Thus, to further explore differences in SNR across conditions, we considered all participants together as one group and compared performance across conditions using post-hoc tests (t-test for paired samples). The data show that SNR for the 26.5-cm disk at 1 m (mean: − 15.98, SD: 2.26) is significantly lower than for the 17.5-cm disk at 1 m (mean: − 11.43, SD: 2.28) (t(30) = 17.128; *p* < 0.001; correlation: 0.787). Furthermore, SNR for the 26.5-cm disk at 1 m is significantly lower than for the 26.5-cm disk at 2 m (mean: − 9.01, SD: 2.83) (t(30) = 13.567; *p* < 0.001; correlation: 0.384) or 26.5-cm at 3 m (mean: − 2.28, SD: 3.23) (t(30) = 29.016; *p* < 0.001; correlation: 0.592), and it is also lower for the 26.5-cm disk at 2 m than at 3 m (t(30) = 12.053; *p* < 0.001; correlation: 0.480). This suggests that the intensity of the emission changes in a systematic way to compensate for the presence of background noise across the different conditions, i.e. the data are generally consistent with the idea that more intense emissions are required to detect weaker echoes in noise.

**Figure 6:**
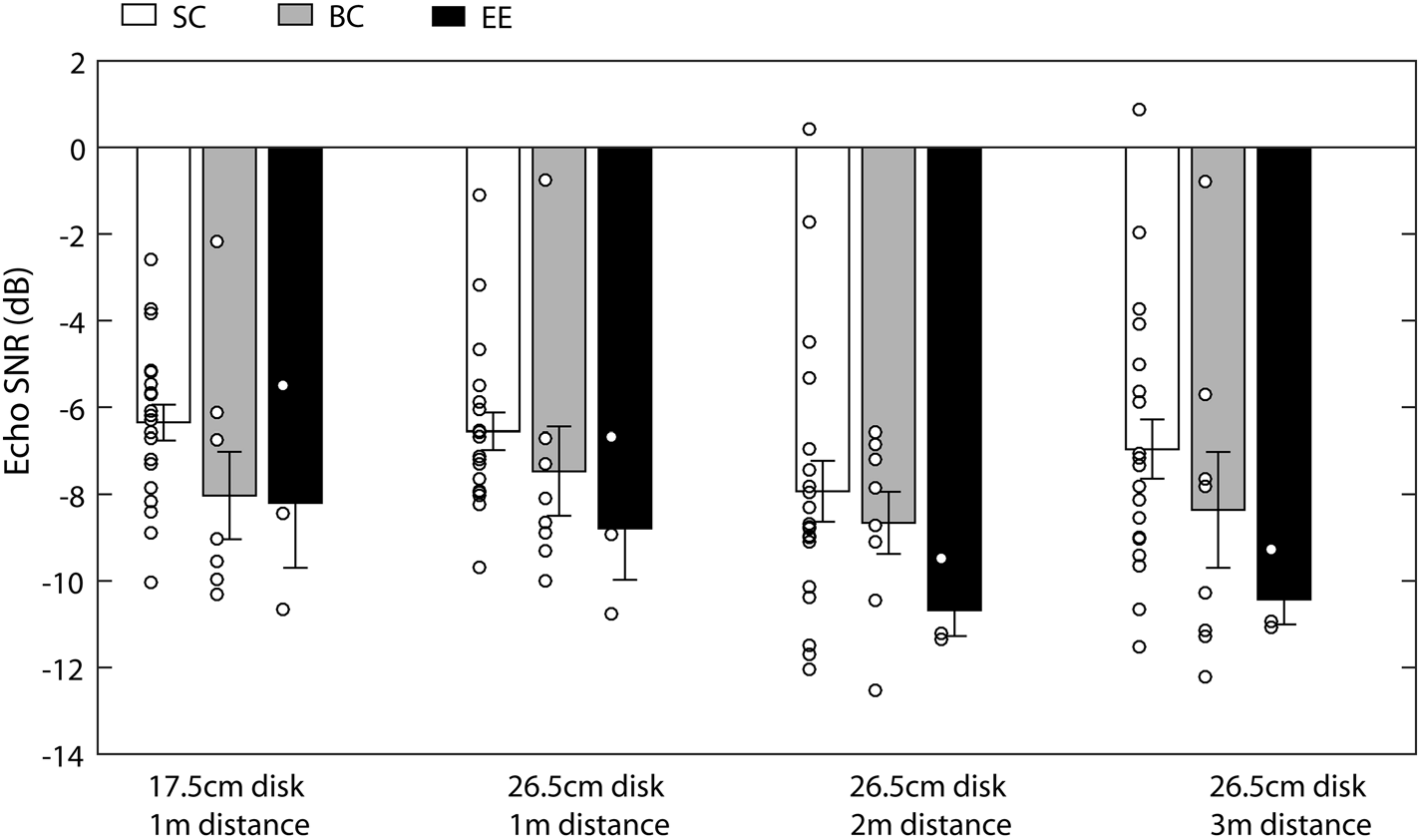
Echo SNRs for the different conditions and participant groups (SC – sighted controls; BC – blind controls; EE – expert echolocators). Bars represent means and error bars represent standard error of the mean across participants. Circles represent individual participants.

Echo SNRs at threshold for the different conditions and groups are shown in Fig. 6. It is evident that echo SNRs are essentially the same for the 17.5 and 26.5-cm disks at 1 m, similar for the 26.5-cm disk at 3 m, and lower for the 26.5-cm disk at 2 m. The effects are similar across participant groups. Consistent with these observations the ANOVA showed a significant main effect of ‘experimental condition’ (F_GG_(2.357, 66) = 2.982; *p* = 0.049; η_p_^2^ = 0.096), whereas the interaction between ‘experimental condition’ and ‘group’ was non-significant (F_GG_(4.714, 66) = 0.364; *p* = 0.862; η_p_^2^ = 0.025) and also the main effect of ‘group’ was non-significant (F(2,28) = 2.522; *p* = 0.098; η_p_^2^ = 0.153). Also the non-parametric Kruskal Walllis test did not reveal any effect of ‘group’ for any of the test conditions (17.5 cm at 1 m: *X*^*2*^(2,31) = 4.790; *p* = 0.091; 26.5 cm at 1 m: *X*^*2*^(2,31) = 5.527; *p* = 0.063 ; 26.5 cm at 2 m: *X*^*2*^(2,31) = 3.261; *p* = 0.196; 26.5 cm at 3 m: *X*^*2*^(2,31) = 4.729; *p* = 0.094). Thus, to further explore differences in echo intensity across conditions, we considered all participants together and compared performance across conditions using post-hoc tests (t-test for paired samples). The data show that echo SNRs do not differ between the 17.5-cm (mean: − 6.96, SD:2.28) and 26.5-cm disks at 1 m (mean: − 7.0; SD: 2.26) (t(30) = 0.161; *p* = 0.873; correlation:0.787), or the 26.5-cm disk at 1 m vs the 26.5-cm disk at 3 m (mean: − 7.66, SD:3.23) (t(30) = 1.385; *p* = 0.176; correlation: 0.592), or the 26.5-cm disk at 2 m (mean: − 8.38; SD: 2.83) vs. 3 m (t(30) = 1.296; *p* = 0.205; correlation: 0.480), but that it was significantly lower for the 26.5-cm disk at 2 m than for the 26.5-cm disk at 1 m (t(30) = 2.681; *p* = 0.012; correlation: 0.384). This suggests that changes in the intensity of the emission yielded similar echo SNRs across conditions, with the exception of lower echo SNR for the 26.5-cm disk at 2 m compared to 1 m, i.e. at a 2-m target distance people don’t need the echo of the 26.5-cm disk to be as loud as at 1 m in order to perform the task.

**Figure 7:**
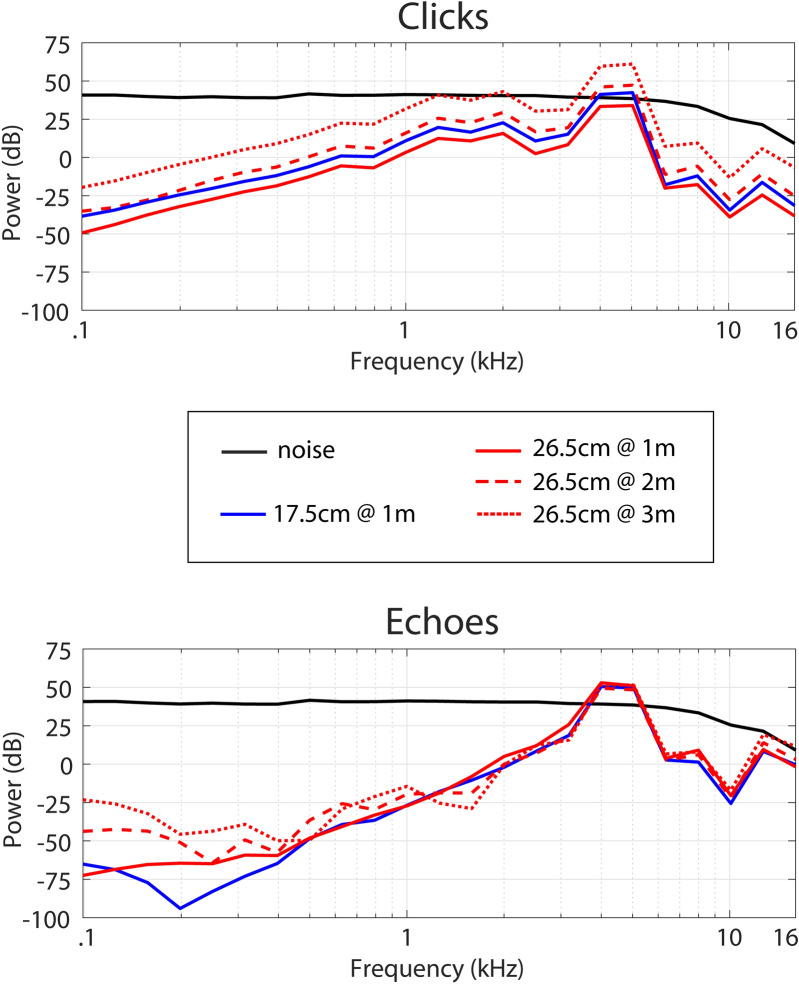
Power spectra (1/3 Octave Bands) for noise and clicks (top panel) and noise and echoes (bottom panel). Black lines denote data for noise, and red and blue solid and dashed lines denote data from clicks and echoes in the various conditions.

SNRs based on overall intensity are negative for both clicks and echoes, which means that emission and echo amplitude are smaller than noise amplitude. Yet, the click had been generated using a center frequency of 4.5 kHz, so that we might suspect that SNR might be positive in the spectrum around a certain frequency band (e.g. 4.5 kHz). Figure 7 shows power Spectra (1/3 Octave Bands) for noise background and click emissions (top panel) and noise background and echoes (bottom panel). It is evident that for clicks as well as echoes spectral power is highest around the 4-kHz and 5-kHz bands, which was expected since the emission used a 4.5-kHz center frequency. Most importantly, while spectral power in those frequency bands changed across conditions for emissions (top panel), it is constant for echoes (bottom panel) and consistently 12 dB above spectral power for noise. This further supports the idea that changes in emission intensity improve or maintain SNRs for echoes, and in our paradigm this happens with respect to power in 4-kHz and 5-kHz frequency bands.

## Discussion

In their entirety the data suggest that people can echolocate in the presence of background noise and suggest that one potential strategy to deal with background noise is to increase intensity of the emission to maintain signal-to-noise ratio of certain spectral components of the echoes with respect to background noise. To this end our study is not only the first to demonstrate that human echolocation works in the presence of background noise, but also the first to suggest that dynamic changes in emission intensity may serve to improve or maintain SNR in human click-based echolocation.

Generally consistent with the idea that the changes in emission intensity serve the function to maintain the SNR of the echo relative to noise, in our study we performed acoustical analyses that showed that (in the overall intensity domain, Fig. 6) for the 17.5-cm disk at 1 m, and 26.5-cm disk at 1 m and 3 m object, SNR was indeed maintained. In contrast, however when the distance of the 26.5-cm object was at 2 m, echo SNR was lower as compared to when it had been presented at 1 m, i.e. people performed the task with echoes of lower intensity relative to the white noise. Most importantly, in the spectral domain (Fig. 7), changes in emission intensity yielded a power of the echo that was consistently 12 dB above power of the noise in 4-kHz and 5-kHz bands, which were the frequency bands in which emissions and echoes had the most power in our paradigm. This strongly suggests that changes in emission intensity serve the function to maintain SNR of echo relative to noise.

With respect to our echo SNR calculations in the overall intensity domain (Fig. 6) and the spectral domain (Fig. 7), although they convey similar information, there are also differences. Namely, the fact that echo SNR when measured by overall intensity level, was significantly lower for the 26.5-cm disk at 2 m than for the 26.5-cm disk at 1 m, whilst by contrast, for all conditions, the 4 to 5 kHz spectral power was constant for echoes and consistently 12 dB above spectral power for noise. This is intriguing and implies that for detection tasks it might possibly be better to measure SNR in the spectral domain, rather than the temporal domain. Luo and colleagues^19^ also suggested that to best understand vocal compensatory behavior in the presence of noise, as well as their neural underpinnings, SNR computations should consider physiological aspects of auditory processing, including the spectral domain. Future work is needed to determine the best way to calculate SNRs for signal detection tasks.

The general effect that people benefit from a rise in the intensity of the emission to increase detection performance can be explained by the non-linear nature of masking. Specifically, even though temporal proximity of clicks and echoes in our study varied across conditions (onset delays of ~ 6 ms, ~ 12 ms and ~ 18 ms), the range of echo onset delays we used implies that detection of echoes will be affected by forward masking (of the echo by the emission)^20,21^ and/or echo suppression^22,23^. Yet, the nonlinear nature of masking makes an increase in click intensity nonetheless a useful strategy to increase detection performance (by increasing SNR)^20,21^. With respect to the range of onset delays we used, however, this nonlinear nature of masking may also imply that different increases in emission intensity may be needed to yield effective echo SNRs at different time delays. The result we found in the overall intensity domain with respect to echo SNRs suggests that this might be the case for the 26.5-cm disk at 2 m as compared to 1 m (i.e. at a time delay of ~ 12 as compared to ~ 6 ms). Yet, echo SNRs at 3 m did not significantly differ from those at either 1 m or 2 m, suggesting that other acoustic factors might also play a role and be linked to different echo SNRs at different time delays. One potential acoustic feature might be for example repetition pitch, where two brief sounds separated by a short gap attain the quality of a single sound carrying a pitch that is inversely related to the duration of the gap^24^. Importantly, the results we found in the spectral domain suggest that the same echo SNR was effective across conditions, i.e. the different changes in emission intensity across conditions yielded the same 12-dB power increase of echo relative to noise in frequency bands of 4 kHz and 5 kHz in all conditions.

We found the same effects in all our participant groups, suggesting that long-term experience does not play a role in the context of our study and the benefits of increasing the intensity of echolocation signals are a basic response of human echo-processing and that these do not rely on long-term training. The task we used required participants to listen to recordings of echolocation sound, instead of making their own clicks. We had chosen this paradigm purposefully to avoid potential confounding effects of differences in spectro-temporal properties of the clicks that people make which are related to performance^12,13^. This raises the possibility, however, that if the task were changed to an active echolocation task where people make their own clicks, expert echolocators who have fine-tuned their clicking skills over many years may show different adaptations as compared to people with less experience. Future work is needed to address this possibility.

Relatedly, studies have described vocal adaptations other than increasing amplitude of speech signals when noise is present. These vocal modifications are referred to as Lombard speech^25–27^. For example, in conjunction with making louder vocalizations, speakers may also produce speech signals with higher spectral frequency content^26,28^. Observations indicate, however, that increasing vocal effort generally leads to more pronounced energy distribution in higher spectral frequencies, so that this effect might be linked to anatomical restrictions with respect to laryngeal sound production rather than intentional signal design^29^. But as observed in human vocal adaptations, echolocating bats also display other vocal modifications to maintain their echolocation behaviour in response to the context^9^. For example, in noisy environments, bats shift the spectral content of their emissions away from that of the masking noise^10,30,31^. They may also change the duration of their emissions in noisy conditions^10,11^ and when their calls are studied in the field as compared to the lab^32^.

The paradigm we used here was chosen to investigate the potential role played by the intensity of the emissions and echoes. There was no flexibility as to other emission aspects such as duration or spectrum. Previous work investigating dynamic adjustments of human echolocation emissions^33,34^ did not employ background noise, but instead manipulated the spatial layout (i.e. target azimuth, size and distance) to investigate dynamics, and they used active tasks where people made their own clicks and could possibly adjust not only intensity but also spectral or temporal aspects. Nonetheless, expert echolocators in those studies only adjusted intensity and number of clicks, suggesting that perhaps in the context of click-based human echolocation intensity is more readily adjustable than spectral or temporal aspects of emissions. It is also possible that different tasks may tap into different dynamics and that for example adjustment of sound pressure is most useful when answering whether or not there is a target. We suggest that future research should investigate these issues further.

Echolocating animals and humans interpret the information that results from the interaction of, generally, self-produced sounds with surrounding surfaces in order to understand the environment. However, organisms who use it to sense their surroundings have demonstrated the need to adjust their acoustic emissions in response to situational challenges as they aim to obtain relevant information about nearby objects. The Lombard effect has described the adaptive vocal responses to noise observed in humans and non-human animals. In bats, one frequent response is the increase in intensity of their echolocation calls when background noise impedes the effective reception of acoustic information^9,10,35^. Changes in the amplitude of the echolocation emission suggest bats’ strategy is to release the signal from the masking effect of the background noise. Bats’ adaptive echolocation behavior in noise was observed in a task where the animals located and landed on a target surface^11^. The authors interpreted bats’ vocal adjustments as an effort to increase the SNR to extract accurate acoustic information of the surroundings when background noise levels interfered with echo perception. Observations indicate that echolocating bats’ adaptive behavior is context-dependent, but this is not only subject to the presence of ambient noise. Echolocating bats adapt the loudness of their emissions when the initial strength of sound reflections is not enough for bats to obtain relevant acoustic information from the surroundings^8^. These observations are consistent with participants’ responses to our task, where we observed that SNRs were dependent on the relevant acoustic information that was obtained from the echolocation signals in each test condition.

Taken together our findings support previous observations of the dynamic nature of human echolocation^33,34^, and provide an important extension as they demonstrate for the first time that people can echolocate in the presence of background noise, and that systematic adjustments of the intensity of emissions enable them to do so. This indicates that for successful echolocation in acoustically dynamic conditions people need dynamic control of the signals that carry relevant acoustic information to support their behaviour. The adjustments that people made to the signals in the present study (i.e. intensity) are comparable to adaptive echolocation behavior of echolocators in active echolocation tasks^33,34^ and might therefore also apply in real-life noisy situations.

## Data Availability

The data generated and analysed during the current study are available from the corresponding author.
